# A Novel Tumor Suppressor Function of Glycine N-Methyltransferase Is Independent of Its Catalytic Activity but Requires Nuclear Localization

**DOI:** 10.1371/journal.pone.0070062

**Published:** 2013-07-30

**Authors:** Suchandra DebRoy, Inga I. Kramarenko, Sampa Ghose, Natalia V. Oleinik, Sergey A. Krupenko, Natalia I. Krupenko

**Affiliations:** Department of Biochemistry and Molecular Biology, Medical University of South Carolina, Charleston, South Carolina, United States of America; University of Saarland Medical School, Germany

## Abstract

Glycine N-methyltransferase (GNMT), an abundant cytosolic enzyme, catalyzes the transfer of a methyl group from S-adenosylmethionine (SAM) to glycine generating S-adenosylhomocysteine and sarcosine (N-methylglycine). This reaction is regulated by 5-methyltetrahydrofolate, which inhibits the enzyme catalysis. In the present study, we observed that GNMT is strongly down regulated in human cancers and is undetectable in cancer cell lines while the transient expression of the protein in cancer cells induces apoptosis and results in the activation of ERK1/2 as an early pro-survival response. The antiproliferative effect of GNMT can be partially reversed by treatment with the pan-caspase inhibitor zVAD-fmk but not by supplementation with high folate or SAM. GNMT exerts the suppressor effect primarily in cells originated from malignant tumors: transformed cell line of non-cancer origin, HEK293, was insensitive to GNMT. Of note, high levels of GNMT, detected in regenerating liver and in NIH3T3 mouse fibroblasts, do not produce cytotoxic effects. Importantly, GNMT, a predominantly cytoplasmic protein, was translocated into nuclei upon transfection of cancer cells. The presence of GNMT in the nuclei was also observed in normal human tissues by immunohistochemical staining. We further demonstrated that the induction of apoptosis is associated with the GNMT nuclear localization but is independent of its catalytic activity or folate binding. GNMT targeted to nuclei, through the fusion with nuclear localization signal, still exerts strong antiproliferative effects while its restriction to cytoplasm, through the fusion with nuclear export signal, prevents these effects (in each case the protein was excluded from cytosol or nuclei, respectively). Overall, our study indicates that GNMT has a secondary function, as a regulator of cellular proliferation, which is independent of its catalytic role.

## Introduction

**Figure 1:**
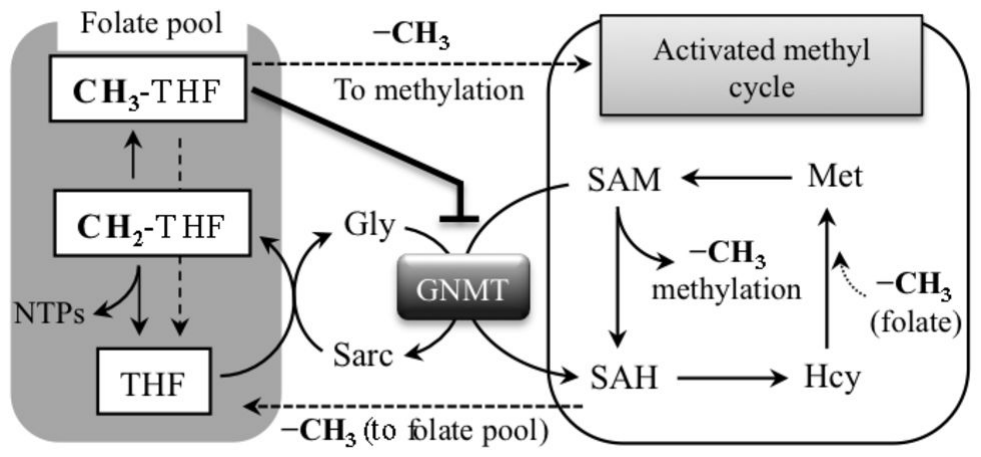
Disposition of GNMT in cellular metabolism. GNMT converts SAM to SAH, methylating glycine to sarcosine. This reaction regulates SAM/SAH ratio and shuttles methyl groups, from activated methyl cycle back to the folate pool. Inhibitory effect of 5-CH3-THF (5-MTHF) on GNMT catalysis is indicated. Hcy, homocysteine; Sarc, sarcosine; THF, tetrahydrofolate.

S-adenosylmethionine (SAM) dependent methyltransferases, a common group of enzymes in the mammalian cell, are involved in numerous reactions of small molecule biosynthesis as well as DNA, RNA and protein methylation [1]. One such enzyme, glycine N-methyltransferase (GNMT), catalyzes the transfer of a methyl group from SAM to the amino group of glycine producing sarcosine (N-methylglycine) and S-adenosylhomocysteine (SAH) [2] (Figure 1). Sarcosine, an intermediate in the pathway of degradation of choline and betaine [3], might be important in the regulation of osmosis and protein stabilization [4,5]. It can be rapidly converted to glycine by folate-dependent sarcosine dehydrogenase [6]. In combination with this reaction, GNMT catalysis constitutes a metabolic cycle, which degrades SAM and simultaneously incorporates the active methyl group into the intracellular folate pool (Figure 1). In this regard, this cycle counterbalances the activated folate methyl cycle, which supplies methyl groups from the folate pool to SAM through the biosynthesis of methionine (Figure 1). It has been proposed that the functional significance of the GNMT reaction is associated with the control of SAM/SAH ratio and the overall methylation potential of the cell [7,8].

GNMT is an abundant enzyme in liver cytosol where it comprises up to 1-3% of the total cytosolic protein [9]. It is also present at high levels in several other tissues [9,10]. An important feature of GNMT catalysis is its regulation by folate, though the reaction itself does not require participation of this coenzyme [11]. An understanding of the regulatory mechanism of GNMT by folate has come from structural studies of the enzyme [12]. In the cell, GNMT is present as a tetramer of four identical subunits. In humans, each subunit is a 32 kDa polypeptide containing 292 amino acid residues [13]. Although each of the four subunits has a catalytic center, the structural organization of the enzyme as a tetramer is important for its catalytic activity. In fact, GNMT dimers or monomers are catalytically inactive [12,14]. The oligomeric structure of the enzyme is also important for its regulation by folate since the GNMT tetramer binds only two molecules of 5-methyltetrahydrofolate (5-MTHF). Each folate molecule binds at the interface formed by two subunits with additional participation of N-termini from two other distant subunits [15]. The folate binding takes place near the entrance to respective SAM binding sites thus restricting conformational changes required for substrate access [15]. In conjunction with the function of GNMT as a reversal route for one-carbon groups from the activated methyl cycle back to the folate pool, this regulation provides an evident feedback mechanism controlling the flow of methyl groups to distinct metabolic pathways [6].

The studies of liver regeneration after partial hepatoectomy in GNMT knockout mice indicated that the protein plays a critical role in hepatocyte viability and proliferation [16]. Of note, the enzyme is strongly down regulated in hepatocellular carcinomas [17–19]. A case-control study revealed that the enzyme is also down regulated in a majority of prostate tumors and has protective effects against this type of cancer [20]. Additional evidence of the role of GNMT in carcinogenesis came from studies of GNMT knock out mice, which revealed a high tendency to develop hepatocellular carcinomas [8,18,20]. These mice also had increased SAM (35-fold or higher) [8,21] as well as increased overall genome methylation including the hypermethylation of promoters of tumor suppressors *RASSF1A* (inhibitor of oncoprotein Ras) and *SOCS2* (inhibitors of JAK/STAT pathway) [22]. The concomitant repression of these genes resulted in the increase of phosphorylated effectors in Ras and JAK/STAT pathways keeping them constantly active [22].

While GNMT is predominantly a cytoplasmic protein, it has been identified as the putative 4S polycyclic aromatic hydrocarbon receptor, a transcriptional activator of cytochrome P-450 1A1 in response to benzo[a]pyren (B[a]P) [23]. Further reports indicated that B[a]P binds to a homodimeric form, which is not a natural one for GNMT but appears after protein phosphorylation [24,25]. GNMT also prevents DNA-B[a]P adduct formation, perhaps by sequestering B[a]P and limiting its active concentration [26,27]. Similar effects of GNMT on DNA-aflatoxin B_1_ adduct formation and reduction of aflatoxin carcinogenicity have been observed [28]. Interestingly, the translocation of GNMT to the nucleus has been reported after binding of B[a]P or aflatoxin, as well as after tetramer dissociation induced by disrupting lysine salt bridges on the monomer interface [14,29]. Though the role of GNMT in the nucleus is not clear at present, the protein is tightly bound to chromatin after nuclear translocation [14].

In the present study, we have observed that GNMT is strongly and ubiquitously down-regulated in human tumors. It is also absent in cancer cell lines, where its expression induces apoptosis through a mechanism associated with nuclear localization of the protein. We are also reporting that the cytotoxic effect of GNMT does not require a catalytically active enzyme, further indicating that the tumor suppressor function of GNMT is not associated with SAM and glycine depletion or sarcosine generation.

## Results

### GNMT is strongly down-regulated in tumor tissues and not expressed in cancer cell lines

**Figure 2:**
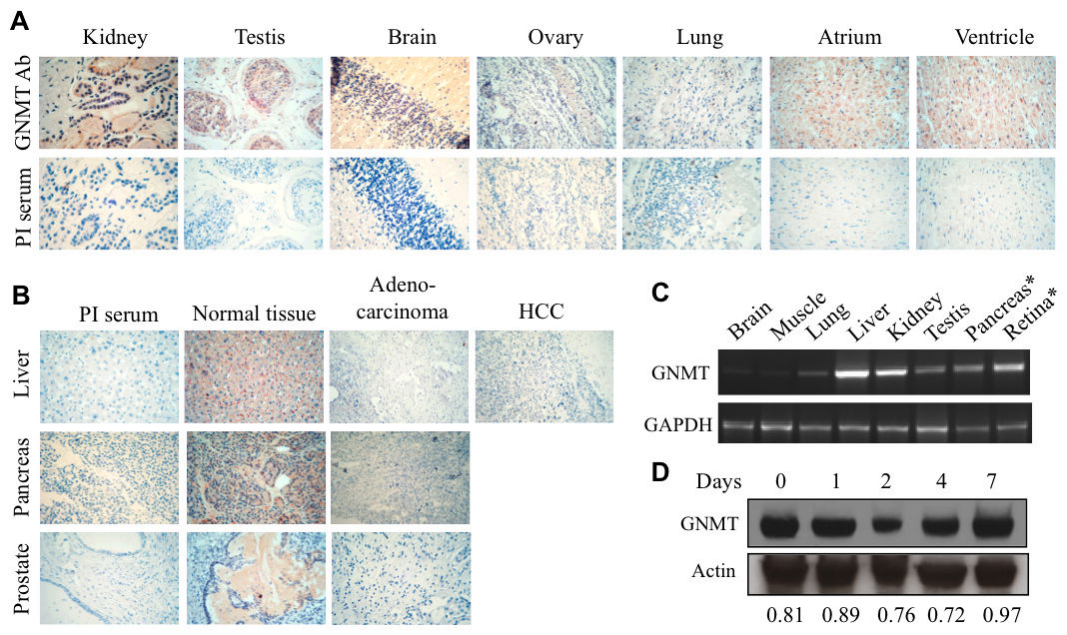
Presence of GNMT in human tissues. **A**. Immunohistochemical staining of normal human tissues with GNMT-specific antibody or preimmune (PI) serum (control). **B**. Immunohistochemical staining of GNMT in normal tissues compared to malignant tumors of the same origin. **C**. Levels of GNMT mRNA in rat tissues; for pancreas, ten-fold less cDNA was used for PCR compared to other tissue; for retina, ten-fold more cDNA was used (in all cases the same amount of cDNA was used for GAPDH amplification). **D**. Levels of GNMT in normal and regenerating rat liver (Western blot of samples obtained 1-7 days after 70% hepatectomy; control sample represents levels of GNMT in resting liver. Numbers indicate ratio of the intensity of GNMT and actin bands.

It has been previously reported that GNMT is an abundant protein in liver, kidney and pancreas [9]. Using an array of human tissues we have demonstrated that GNMT is also expressed in other tissues including prostate, heart, brain, testis, ovary and lung (Figure 2, A,B). Levels of GNMT mRNA analyzed in a panel of rat tissues were in concordance with tissue distribution pattern of the protein in humans (Figure 2C). Our experiments have further revealed the loss of GNMT in tumors originated from several types of human tissues (Figure 2B). Similar to tumor tissues, GNMT was not detectable at the protein level in several human cancer cell lines (data not shown). The following common cell lines were examined in this study: PPC-1, PC-3, Tsu-Pr1, HepG2, A549, PANC-1. To explore whether GNMT down regulation is associated with malignant phenotype or whether it could be a characteristic of proliferating cells, we evaluated levels of the protein in regenerating liver tissue. After 70% hepatoectomy, liver rapidly restores its size due to enhanced hepatocyte proliferation, with DNA synthesis reaching peak between 24 and 48 h after surgery [30]. We have observed that levels of GNMT in regenerating liver were similar to those in normal liver (Figure 2D), which indicates the lack of direct association between cellular proliferation and GNMT levels. In line with this observation, the protein is present in rapidly proliferating NIH3T3 fibroblasts (data not shown). Interestingly though, we did not detect GNMT in non-cancer origin HEK293 cells (see below).

### Re-expression of GNMT in cancer cells inhibits proliferation

**Figure 3:**
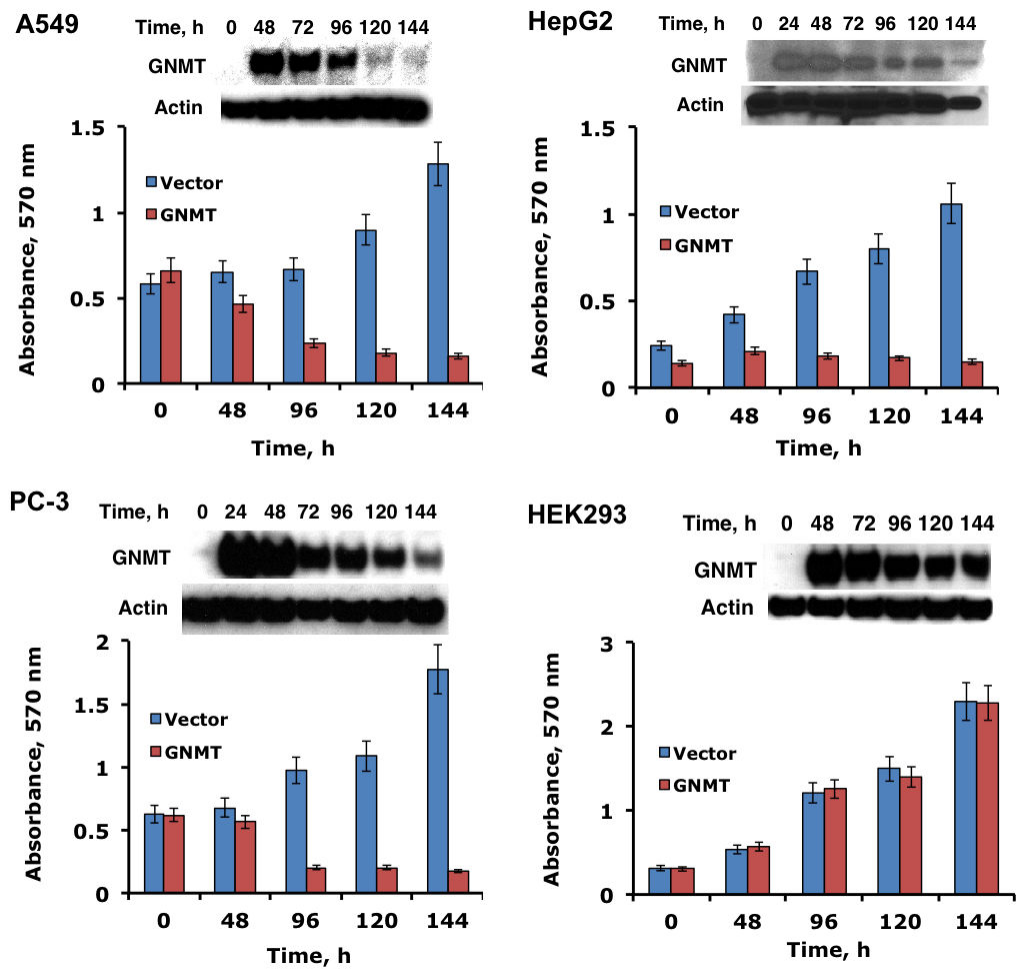
Effect of GNMT transient transfection on cellular proliferation. Cell viability was assessed by MTT assay (absorbance at 570 nm reflects the number of live cells). *Error bars* represent ± S.D., *n =3*. Insets show levels of GNMT (Western blot) at different time points after transfection.

To investigate effects of GNMT on proliferation of cancer cells, three human cell lines (A549, a lung non-small cell carcinoma; PC-3, a prostate adenocarcinoma; and HepG2, an hepatocarcinoma) were transiently transfected with a pcDNA3.1/GNMT construct. Immunoblot assays indicated the appearance of GNMT 24 h post-transfection in all cell lines; highest levels were achieved at 24-72 h post-transfection (Figure 3). GNMT remained at detectable levels up to five days after a single round of transfection indicating a long half-life (Figure 3). The MTT assay has further shown that GNMT expression induced strong antiproliferative effects in all three studied cancer cell lines. At the same time, a transient expression of GNMT in non-cancer origin HEK293 cells (immortalized human embryonic kidney cell line) did not produce noticeable effects on proliferation (Figure 3).

### GNMT induces apoptosis in the absence of DNA damage but does not affect the cell cycle

**Figure 4:**
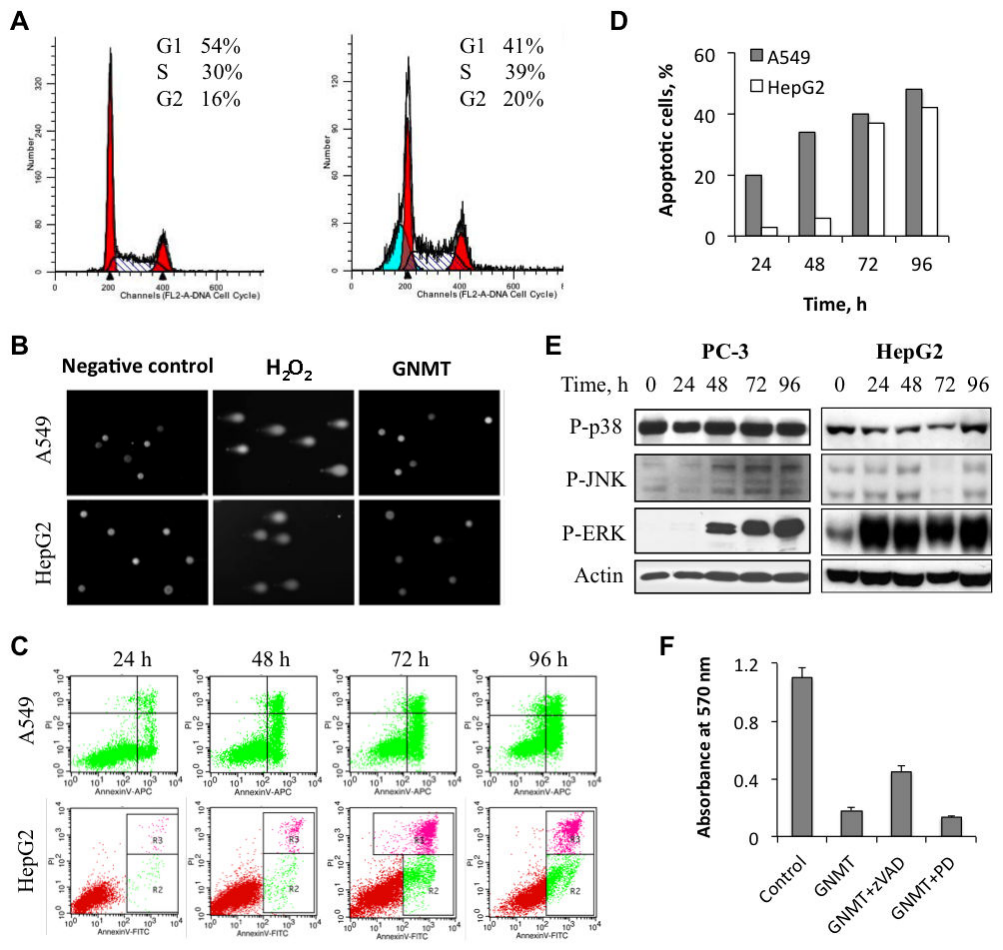
Cellular responses to GNMT expression. A. Distribution of GNMT-expressing cells (*right panel*) between cell cycle phases (propidium iodide staining) compared to control (*left panel*) GNMT-deficient cells. **B**. Assessment of DNA damage in GNMT expressing cells by the Comet assay. **C**. Apoptotic cells assessed by Annexin V/propidium iodide staining after GNMT expression (*bottom right quadrant*, early apoptotic cells; *upper right quadrant*, late apoptotic cells); only green cells (expressing GFP-GNMT) were evaluated. **D**. Calculation of apoptotic cells from C. **E**. Activation of ERK phosphorylation in response to GNMT expression. **F**. zVAD-fmk, but not ERK inhibitor PD98059, partially rescues cells from the antiproliferative effect of GNMT (data for A549 cells are shown).

To study whether antiproliferative effects of GNMT are associated with the cell cycle arrest, we performed cell cycle analysis using propidium iodide staining. These experiments did not show evidence of cell cycle arrest upon GNMT expression, but did show a prominent sub-G1 peak indicating that GNMT induces apoptosis (Figure 4A). The apoptosis was not associated with DNA damage as was judged from the comet assay (Figure 4B). This assay evaluates single and double-stranded DNA breaks based upon the ability of denatured, cleaved DNA fragments to migrate out of the nucleoid under the influence of an electric field. No DNA damage upon GNMT expression in A549 cells was observed in these experiments compared to cells positive for comet tails (Figure 4B). The annexin V assay has shown that about 20% of A549 cells became apoptotic at 24 h GNMT post-transfection with a significant increase in the number of apoptotic cells at later time points (Figure 4, C and D). The apoptotic effect of GNMT was less prominent at early time points in HepG2 cells, but reached about the same levels at 72 and 96 h post-transfection (Figure 4, C and D).

We have also observed that GNMT expression resulted in the activation of one of the MAP kinases, ERK: its strong phosphorylation was seen upon GNMT expression (Figure 4E). Phosphorylation levels of two other MAPKs, p38 and JNKs, were not affected in our experiments (Figure 4E). In the case of GNMT, this activation was a pro-survival response since the treatment of cells with ERK inhibitor PD98059 did not rescue cells but enhanced GNMT-induced cell death (Figure 4F). In contrast, treatment of GNMT-expressing cells with pan-caspase inhibitor zVAD-fmk partially reversed the proliferation inhibitory effect (Figure 4F). Interestingly, activation of ERK was seen beginning 24 h GNMT post-transfection, while profound antiproliferative effects took place at later time points (after 72 h). Thus, it is possible that the antiproliferative effect of GNMT was observed at later time points due to the interference from ERK pro-survival action.

### Catalytically inactive GNMT mutants retain the antiproliferative effect

**Figure 5:**
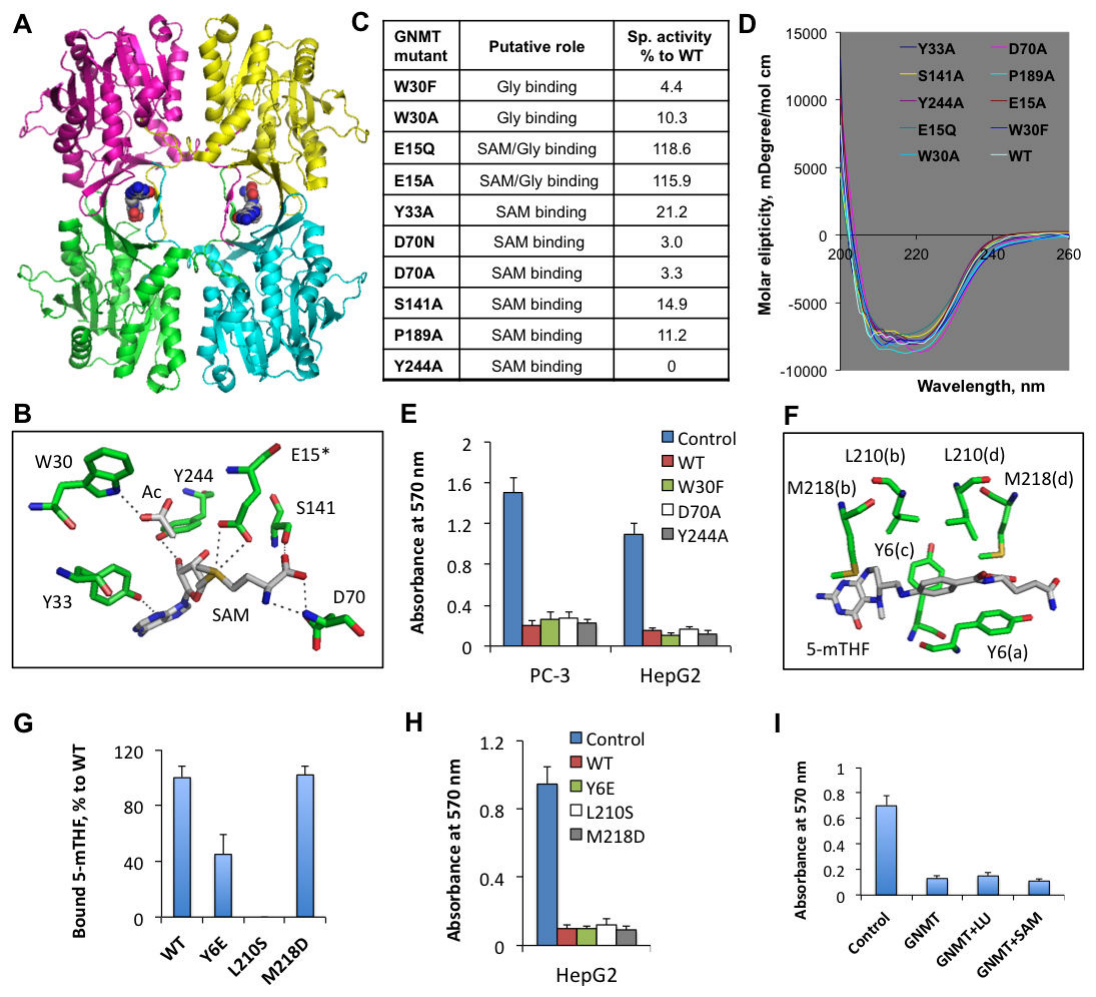
Catalytically inactive or folate-binding deficient GNMT mutants are capable of the antiproliferative effect. **A**. Crystal structure of GNMT tetramer (RCSB Protein Data Bank 3ths; subunits are shown in different colors) with bound 5-MTHF monoglutamate (two molecules shown in spacefill mode are bound per tetramer). **B**. Positions of amino acid residues in the GNMT catalytic center (from RCSB Protein Data Bank 1XVA). Acetate (Ac) is the competitive inhibitor of Gly and presumably occupies the same position in the active center. Glu 15 (E15*) is from a different subunit. Dotted lines indicate hydrogen bonds. **C** and **D**. The enzyme activities and CD spectra of GNMT mutants, analyzed in this study. **E**. The MTT proliferation assay of cells transfected with empty vector (control), wild type GNMT (WT), or corresponding mutants. *Error bars* represent ± S.D., *n =3*. **F**. Folate binding site at the GNMT subunit interface (as shown in panel **A**); Selected for mutagenesis are residues within close distance to 5-MTHF molecule (these residues are from all four subunits, which are denoted in parentheses). **G**. Binding of 5-MTHF by GNMT mutants. *Error bars* represent ± S.D., *n =2*. **H**. The MTT proliferation assay of cells transfected with empty vector (control), wild type GNMT (WT), or folate-binding deficient mutants mutants. *Error bars* represent ± S.D., *n =3*. **I**. The supplementation with excessively high media folate or SAM does not rescue cells from the GNMT antiproliferative effect. *Error bars* represent ± S.D., *n =3*.

In the GNMT tetramer, each subunit has a catalytic center, but only two folate-binding sites per tetramer are formed (Figure 5A) [15]. To determine whether catalytically active GNMT is required for the induction of antiproliferative effects, we have generated an array of GNMT mutants. Targets for the mutagenesis were selected based on the arrangement of the residues within the catalytic center: residues within the hydrogen bond distance to SAM or acetate (the latter occupies the position of the second substrate, glycine) were interrogated (Figure 5B). These experiments have identified several residues, crucial for the catalysis (Figure 5C). All studied mutants retain a typical for the wild type protein tetrameric structure, which was assessed by size-exclusion chromatography (data not shown). CD spectra of the mutated proteins were almost identical to that of the wild type enzyme (Figure 5D), indicating that the lack of activity was not due to protein misfolding or extensive conformational changes.

Three of the mutants, one with undetectable catalytic activity (Y244A) and two with a dramatically reduced activity (D70A and W30F) (Figure 5C), were tested for their antiproliferative properties. MTT proliferation assays of HepG2 or PC-3 cells transfected with corresponding mutants demonstrated that enzymatically inactive GNMT retains the full ability to induce cytotoxic effects: no difference in the antiproliferative activity, as compared to the wild type enzyme, was observed (Figure 5E). These data suggest that the antiproliferative effect of GNMT is independent of its catalytic activity.

### Antiproliferative effects of GNMT are folate-independent

GNMT enzymatic function strongly depends on intracellular levels of reduced folate [2] because binding of 5-MTHF pentaglutamate inhibits the enzyme [11]. To study whether the folate-binding ability is required for the antiproliferative effect of GNMT, we searched for mutants which would lack this function. Analysis of the folate binding site showed that three residues, Tyr 6, Leu 210 and Met 218, are in close proximity to the folate molecule bound at the subunit interface (Figure 5F), indicating their potential involvement in the ligand binding. Folate binding assays of respective GNMT mutants revealed that Leu 210 is strictly required for folate binding (Figure 5G). Replacement of Tyr 6 also decreased the folate binding ability of the protein to about 45% of the wild type GNMT, while replacement of the methionine did not produce a notable effect (Figure 5G). Of note, Y6E and M218D mutants fully retained their tetrameric structure as was determined by size-exclusion chromatography on Sephacryl S300. The chromatography of the L210S mutant revealed the presence of three oligomeric forms: tetrameric (about 40% of the total mutated protein); dimeric (about 50%); and monomeric (about 10%). MTT proliferation assays have further shown that all three mutants inhibit proliferation similar to the wild type enzyme (Figure 5H). Thus, the inability to bind folate did not prevent the antiproliferative effect of GNMT.

To further confirm the folate-independent antiproliferative effect of GNMT, we studied whether it can be reversed by high folate supplementation. We have grown GNMT-expressing cells on media supplemented with 10 μM reduced folate leucovorin (5-formyl-THF or folinic acid) in addition to a typical supplementation with 2.2 µM folic acid. In the cell, leucovorin is rapidly incorporated into the overall pool of reduced folates: a significant elevation of the total reduced folate (4-fold), including more than a 5-fold increase of 5-MTHF, has been observed in A549 cells upon such supplementation [31]. Furthermore, it also protected cells against cytotoxic effects of another folate regulatory enzyme, ALDH1L1 [32]. Our experiments have demonstrated that leucovorin did not rescue GNMT-expressing cells (Figure 5I). We have also demonstrated that the GNMT cytotoxic effect cannot be alleviated by supplementation with SAM (Figure 5I). Thus, we conclude that this effect proceeds through a mechanism independent of the metabolic function of the enzyme.

### GNMT is localized to nuclei

**Figure 6:**
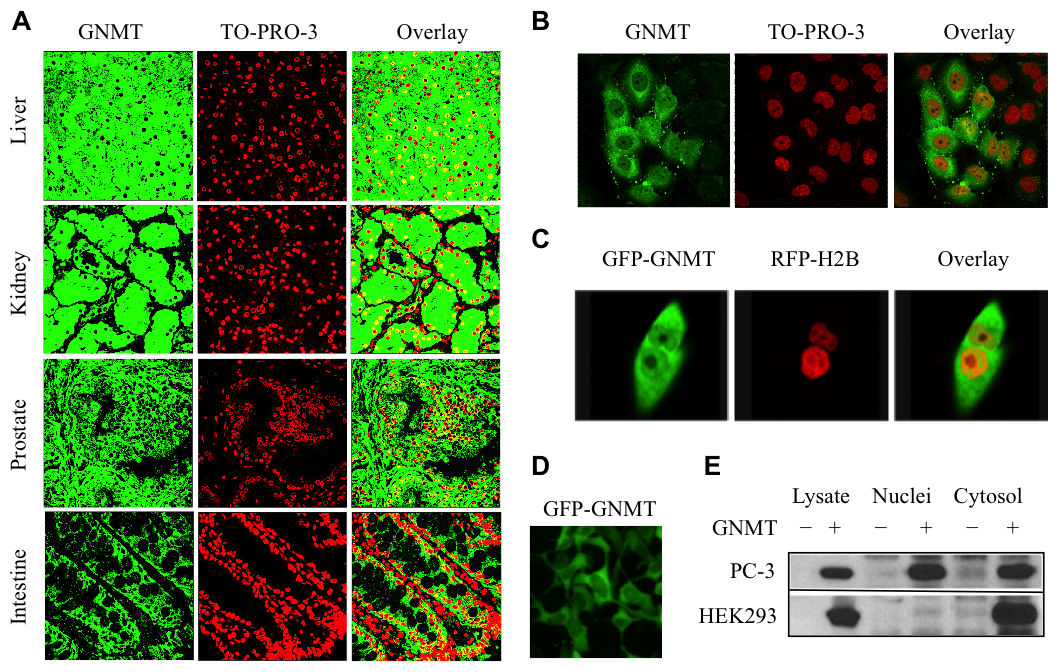
GNMT is localized to nuclei. **A**. Detection of GNMT in a panel of normal human tissues by immunohistochemical staining and confocal microscopy (*left panel*, GNMT (*green*) detected by immunostaining with anti-GNMT antibody and secondary antibody conjugated with Alexa Fluor 488 dye; *middle panel*, nuclei (*red*) stained with To-Pro-3 dye; *right panel*, overlay with *yellow* indicating co-localization). **B**. Nuclear localization of GNMT in fixed PC3 cells assessed by confocal microscopy: *left panel*, GNMT (*green*) detected by immunostaining with anti-GNMT antibody and secondary antibody conjugated with Alexa Fluor 488 dye; *middle panel*, nuclei (*red*) stained with To-Pro-3 dye; *right panel*, overlay with *yellow* indicating co-localization. **C**. Accumulation of GNMT in nuclei monitored by confocal microscopy in live cells: *left panel*, GFP-GNMT (*green*); *middle panel*, RFP-H2B (*red*); right panel, overlay with *yellow* indicating co-localization. **D**. Exclusion of GFP-GNMT from nuclei in HEK293 cells detected by fluorescence microscopy. **E**. Detection of GNMT in nuclear and cytosolic fractions of PC-3 and HEK239 cells 96 h post-transfection. Subcellular fractions were obtained by differential centrifugation. Control (-) or GNMT transfected (+) cell fractions are shown.

We have performed histochemical staining of GNMT in a panel of human tissues to analyze the sub-cellular localization of the protein. In these experiments, positive staining for GNMT in nuclei has been seen (Figure 6A). While the distribution of GNMT-positive nuclei was not even throughout the cross-section of a specific tissue and further varied between different tissues (Figure 6A), the nuclear localization of the endogenous protein in normal tissues was evident. Similar to the observation of the nuclear localization of GNMT in human tissues, transiently expressed GNMT was localized to nuclei in PC-3 cells (Figure 6B). This finding was confirmed in experiments with live cells, which were transfected with GFP-GNMT fusion: the co-localization of the construct (green color) with RFP-H2B fusion (localizes to the nuclei yielding red color) was seen by the confocal microscopy (Figure 6C). Interestingly, the exclusion of the enzyme from the nuclei was seen in HEK293 cells, the cell line resistant to GNMT cytotoxic effects (Figure 6D). Translocation of transiently expressed GNMT into nuclei of PC-3 cells, but not HEK293 cells, was also confirmed by Western blot assays after subcellular fractionation (Figure 6E).

### Exclusion of GNMT from the nucleus protects cells from antiproliferative effects

**Figure 7:**
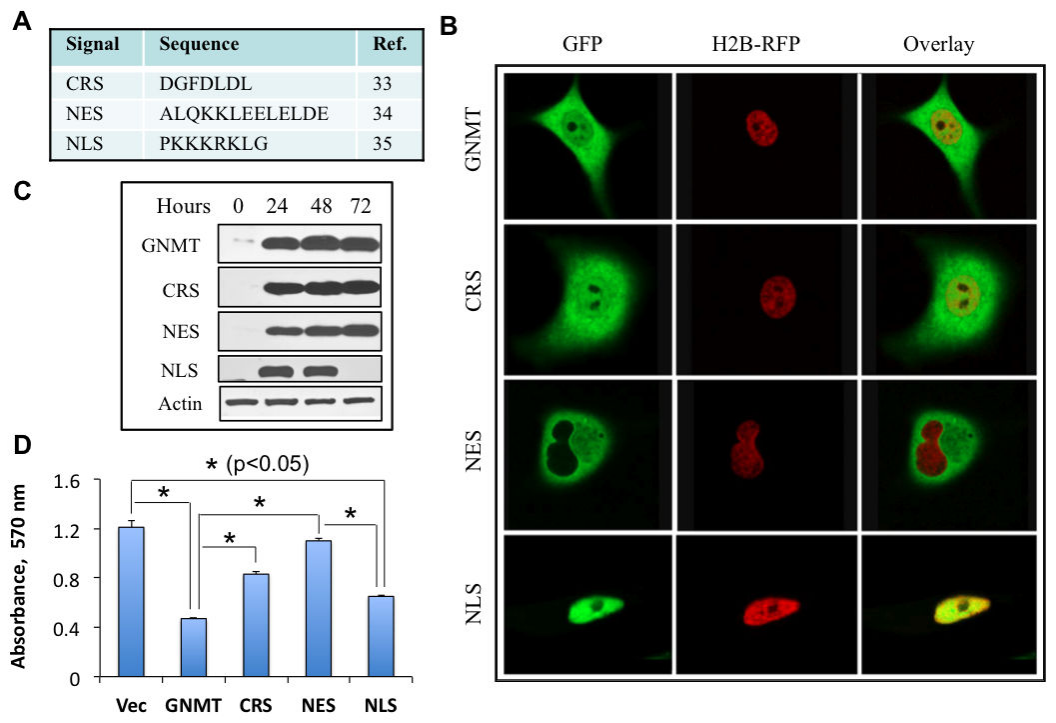
Subcellular localization-specific effects of GNMT. **A**. Sequences used to target GNMT to cytosol or nucleus. B. Distribution of GNMT fusion constructs between cytosol and the nucleus. All constructs included GFP tag at the C-terminus of GNMT. Respective subcellular targeting sequences were introduced at the C-terminus of the GFP tag. **C**. Levels of corresponding GNMT constructs after transient transfection (Western blot assay). **D**. MTT assay of cells transfected with GNMT constructs. *Error bars* represent ± S.D., *n =2*; *, *p < 0*.*05*.

To study whether subcellular localization of GNMT defines its cytotoxic effects, we have generated GFP-GNMT fusion constructs, which also had cytoplasm retention signal of PTEN (CRS), nuclear export signal of MAPKK (NES), or nuclear localization signal of SV40 large T antigen (NLS) (Figure 7A) [33–35]. Enzyme assays of the fusion proteins have revealed that they all retained full catalytic activity and folate binding similar to the wild type enzyme. Confocal microscopy has shown that, as expected, two of these constructs, with NLS or NES, resided exclusively in the nucleus or cytoplasm, respectively (Figure 7B). The CRS did not restrict GNMT exclusively to the cytoplasm and its presence in the nuclei was still evident (Figure 7B). Western blot assays indicated that the expression levels of all constructs were very similar (Figure 7C). Interestingly, GNMT localized to the nucleus still exerted a strong antiproliferative effect, while exclusion of the protein from nuclei rescues cells (Figure 7D). GNMT bearing CRS also induced the antiproliferative effect, although to a lesser extent than the wild type protein (Figure 7E), which is in agreement with the partial exclusion of the protein from nuclei.

## Discussion

In the present study, we have investigated the effect of GNMT, a folate regulatory enzyme, on cancer cells. GNMT is not directly involved in the conversion of folate coenzymes but its catalytic activity is regulated by 5-MTHF, the most common natural folate [2]. Folate metabolism is typically enhanced in cancer cells because of its key role in the *de novo* nucleotide biosynthesis and methylation processes [36]. Accordingly, to support the increased flow of one-carbon groups towards biosynthetic processes through folate pathways, folate enzymes are often elevated in cancers. Examples of this trend include dihydrofolate reductase, the folate receptor and mitochondrial folate enzymes of the serine/glycine metabolism [37–40]. In contrast to these enzymes, in our study cancer tissues and tumor-derived cell lines demonstrated the total loss of GNMT. Furthermore, we have shown for the first time that GNMT exerts a direct inhibitory effect on cancer cells by inducing apoptosis. Interestingly, in these experiments non-cancer origin HEK293 cells were resistant to GNMT. In line with this finding, GNMT is present in rapidly proliferating normal fibroblasts (NIH3T3 cells). These findings suggest that the effect of the enzyme might not be relevant to cellular proliferation in general. Further support of this conclusion came from studies of regenerating liver, which still maintains high levels of GNMT upon rapid hepatocyte proliferation. Thus, the overall relationship between GNMT and cancer is reminiscent of another folate regulatory enzyme, ALDH1L1, which is also silenced in tumors and exerts strong antiproliferative effects in cell culture models [32].

The inhibitory effect of GNMT on cancer cells perhaps explains the tendency of tumors to eliminate this protein. Apparently, cancer cells recognize its presence as a stress judging from the activation of ERK in response to the enzyme expression. While ERK activation can be either a pro-apoptotic or pro-survival response [41], in the case of GNMT it is the latter one since the treatment of cells with the ERK inhibitor PD98059 in our study enhanced cell death upon GNMT expression. Of note, the association between GNMT and ERK, with an increase of phospho-ERK levels in GNMT knockout mice and liver tumors developing in these mice, has been previously observed [42]. In this published study, the steady-state levels of phospho-ERK were measured, whereas we have detected a much stronger and rapid increase in phospho-ERK which is indicative of stress response. Surprisingly, our finding is in disagreement with two recently published studies, which demonstrated that GNMT has a promoting effect on cancer cell proliferation and that knockdown of the enzyme by RNA interference induces apoptosis and inhibits tumor growth [43,44]. Furthermore, in the above studies GNMT levels were detected by immunoblot assays in several prostate cancer cell lines as well as in human prostate tumors, which was opposite to our observations. While we cannot offer a suitable explanation of this controversy, our data are very much in-line with reports showing drastically decreased GNMT in more than 80% of prostate cancer tissues [20] as well as in liver cancers [17]. In addition, analysis of the RefExA microarrays database indicated very low levels of GNMT mRNA in numerous cancer cell lines, including prostate cancer cells, a phenomenon that further questions the proliferation-promoting role of GNMT.

As a regulatory enzyme, GNMT balances the flow of methyl groups toward methylation processes by utilizing SAM, the universal methyl donor in the cell. For example, it is required for the biosynthesis of polyamines, which are essential compounds for cellular function. In fact, cancer cells require high supplies of polyamines with up to 70% of SAM directed to this biosynthesis [45,46]. Therefore, down regulation of GNMT in cancers could be explained by the necessity to support high levels of SAM. Alternatively, a possibility that the enzyme would interfere with cell proliferation by a different mechanism should not be ignored. One such mechanism could be associated with limiting intracellular glycine, which is converted to sarcosine by GNMT catalysis. Indeed, several recent studies have underscored the importance of the glycine metabolism for cancer cells [39,40]. For example, the elevation of glycine decarboxylase, a component of the mitochondrial glycine cleavage system, is associated with high growth rate and tumorigenic potential in tumor initiating cells [40]. In fact, the over-expression of this enzyme promotes cellular transformation, and its aberrant activation inversely correlates with survival in lung cancer patients [40]. Another study reported that levels of SHMT2, a mitochondrial enzyme producing glycine from serine, correlated with enhanced proliferation of cancer cells, while the increased expression of this enzyme was associated with greater mortality in breast cancer patients [39].

The role of GNMT in the regulation of sarcosine levels could be another mechanism of the enzyme involvement in tumorigenesis. While sarcosine does not have a clear function in the cell, it has been suggested as a biomarker of aggressive prostate tumors in humans and an indicator of increased invasiveness [47]. Our findings - that catalytically inactive GNMT mutants still evoked the same antiproliferative effect as the wild type enzyme - indicate that the suppressor effects of GNMT were not caused by depletion of SAM/glycine or sarcosine production. Hypothetically, if the GNMT catalytic role is detrimental for accelerated proliferation, the selection of catalytically inactive mutants upon malignant transformation would be expected. Of note, such naturally occurring mutants of GNMT with drastically reduced catalytic activity have been reported, though they are extremely rare and are not associated with cancer [48–50]. The lack of such selection argues in support of catalytically independent effects of the protein on proliferation. In line with this hypothesis, mutants lacking the ability to bind 5-MTHF were also active towards the induction of apoptosis. This finding indicates that the antiproliferative effect of GNMT is not associated with the restriction of availability of the intracellular folate. In agreement with this conclusion, the elevation of media folate did not reverse the cytotoxic effect of GNMT.

Our study offers a mechanism for the effects of GNMT on cancer cells, which is catalysis-independent but associated with nuclear localization of the protein. Indeed, while GNMT is predominantly a cytoplasmic protein, its localization in nuclei and mitochondria has been reported [29,43,51]. Likewise, in our experiments GNMT was present in nuclei of normal human tissues and transfected cells. Importantly, the observation that nucleus-targeted GNMT, totally excluded from the cytosol, evokes the antiproliferative effect to the same extent as normal GNMT suggests the mechanism associated with nuclear function. In further support of this conclusion, the exclusion of GNMT from nuclei (by tagging it with NES) protected cells from the antiproliferative effect of the protein. Interestingly, a recent study has implied that the cytoplasmic localization of GNMT is associated with lower survival of prostate cancer patients [43]. This observation is in line with our data demonstrating that GNMT non-sensitive HEK293 cells exclude the enzyme from the nucleus.

We propose that the function of GNMT in the nucleus is associated with its interaction with specific nuclear proteins but not with its catalysis. Of note, GNMT has been shown to interact with Niemann-Pick type C2 protein in liver cytosol to regulate cholesterol homeostasis [52]. We further propose that nucleus-localized GNMT serves as either co-activator or co-repressor of gene transcription.

## Materials and Methods

### Immunohistochemical staining

Immunohistochemical staining was performed as described elsewhere [32] using in-house GNMT-specific polyclonal antibody (1:1000) generated against the full-length recombinant human enzyme. In the control experiments, pre-immune serum was used in the same procedure. All slides were counterstained with hematoxylin. The tissue sections were examined using an Axioskop 20 upright microscope (Carl Zeiss, Oberkochen, Germany).

### Cell culture, transfection and viability assays

Cell lines were obtained from American Type Culture Collection. Cell culture media and reagents were purchased from Invitrogen. Cells were maintained in RPMI 1640 supplemented with 10% fetal bovine serum, 2 mM glutamine and 1 mM sodium pyruvate (complete medium) at 37 ^o^C under humidified air containing 5% CO_2_. Cell viability was assessed by MTT cell proliferation assay using cell Titer 96 kit (Promega). In this assay the absorbance at 570 nm reflects the number of live cells. Transfection with pcDNA3.1/GNMT or pcDNA3.1/GNMT-GFP vectors (2 µg) was performed using 1.4 x 10^6^ cells and Amaxa electroporation kit ‘V’ according to the manufacturer’s directions. To select transfected cells, 500 μg/ml of G418 (Sigma) was added to the culture 24 h post-transfection. The ERK inhibitor PD98059 and zVAD-fmk were from Calbiochem.

### Immunoblot analysis

Western blot analysis was performed using cell lysates prepared in buffer containing 50 mM Tris-HCl buffer (pH 8.0), 150 mM NaCl, 2 mM EDTA, 1% Triton X-100, 0.1% SDS, 1 mM dithiothreitol, 1 mM PMSF and protease inhibitor cocktail (Sigma). Cell lysates were subjected to SDS-PAGE followed by immunoblot with corresponding antibodies.

### Antibodies

GNMT was detected using in-house GNMT-specific polyclonal antiserum (1:10,000); actin was detected using monoclonal antibodies (1:5000, Calbiochem). Phos-SAPK/JNK (Thr183/Tyr185), Phospho-ERK1/2 (Thr202/Tyr204), Phospho-p38 (Thr180/Tyr182) were detected using antibodies from Cell Signaling Technology (1:1000).

### Apoptosis assay and cell cycle analysis

Apoptosis was detected by Annexin-V labeling using Annexin-V-FLUOS staining kit (Roche Molecular Biochemicals, Indianapolis, IN). Only green cells (expressing either GFP or GFP-GNMT) were analyzed for Annexin-V staining. For the cell cycle analysis, cells were fixed in 40% ethanol and stained in 38 mM sodium citrate buffer containing 50 mg/ml propidium iodide, 0.1% Triton X-100 and 7 Kunitz units/ml RNase A. Flow cytometry analysis was performed in the Hollings Cancer Center (HCC) Flow Cytometry Core facility on a Becton Dickinson FACSCalibur using CellQuest and Mod Fit Software.

### Comet assay

DNA strand breaks were assessed using an Alkaline Comet, Assay kit (Trevigen) following manufacturer’s protocols. Images were captured using a Zeiss Axio Observer D1 multichannel fluorescent microscope.

### Protein expression, purification and mutagenesis

Recombinant GNMT was expressed in *E. coli* and purified by several subsequent chromatographic steps as we previously described [14]. Site-directed mutagenesis was carried out using a QuickChange site-directed mutagenesis kit (Stratagene) and confirmed by DNA sequencing of the mutant constructs at the Nucleic Acid Analysis Core Facility, MUSC.

### Enzyme activity and folate binding assays

GNMT activity was measured by using ^3^H-radiolabeled SAM and acid-washed charcoal separation method as described [53]. Binding of 5-MTHF to GNMT was evaluated using the separation of the unbound ligand on centrifugal filter cartridges followed by spectrophotometric measurements [54].

### Confocal microscopy

Cells grown in Lab-Tek II Chamber (Nulg Nunc International) were fixed with 3.7% of methanol-free formaldehyde for 10 min, and permeabilized with 0.1% Triton X-100 for 5 min. After blocking with 10% pre-immune goat serum in PBS for 45 min slides were incubated with GNMT-specific antibody (1:10,000 dilution) overnight at 4 ^o^C. This was followed by incubation with secondary goat anti-rabbit antibody conjugated with Alexa Fluor 488 (Molecular Bioprobes) in dark chamber for 45 min at room temperature. Nuclei were stained by To-Pro-3 iodide (Invitrogene) according to manufacturer’s protocol. Tissue sections prepared as described above for the immunohystochemical staining were visualized by confocal microscopy using the same procedure as for fixed cells. For live imaging, cells were transfected with the pEGFP vector bearing GFP-GNMT fusion construct (GFP was engineered at the C-terminus of GNMT). To image nuclei in live cells, twenty-four hours later cells were transfected with CellLight Histone 2B–RFP (BacMam 2.0, Molecular Probes). Images were captured and processed using a Leica TCS SP2 AOBS scanning confocal microscope in the HCC Cell and Molecular Imaging Core Facility.
